# The impact of nutritional intervention on quality of life and outcomes in patients with head and neck cancers undergoing chemoradiation

**DOI:** 10.3389/fonc.2024.1475930

**Published:** 2024-10-21

**Authors:** Sara Cardellini, Chiara Lucrezia Deantoni, Matteo Paccagnella, Amanda Casirati, Andrea Pontara, Alessandro Marinosci, Moreno Tresoldi, Leone Giordano, Anna Chiara, Italo Dell’Oca, Nadia Gisella Di Muzio, Riccardo Caccialanza, Aurora Mirabile

**Affiliations:** ^1^ Clinical Nutrition, IRCCS San Raffaele Scientific Institute, Milano, Italy; ^2^ Department of Radiation Oncology, IRCCS San Raffaele Scientific Institute, Milano, Italy; ^3^ Translational Oncology ARCO Foundation, Cuneo, Italy; ^4^ Clinical Nutrition and Dietetics Unit, Fondazione IRCCS Policlinico San Matteo, Pavia, Italy; ^5^ General Medicine and Advanced Care Unit, IRCCS San Raffaele Scientific Institute, Milano, Italy; ^6^ Vita-Salute San Raffaele University, Milano, Italy; ^7^ Department of Otorhinolaryngology, IRCCS San Raffaele Scientific Institute, Milano, Italy

**Keywords:** head and neck cancer, nutritional counselling, oral nutritional supplement, quality of life, body composition, high-dose cisplatin, chemotherapy, radiotherapy

## Abstract

**Introduction:**

Chemoradiotherapy in head and neck cancer patients has a curative intent but often deteriorates nutritional status leading to sarcopenia and cachexia.

**Methods:**

In this observational and single-centered study, a prospective evaluation of several biochemical and anthropometrical parameters, weight loss, handgrip strength, visual analogue scale of appetite, questionnaires associated with malnutrition & quality of life and body composition (obtained by Bioelectrical Impedance Vector Analysis) was performed before and after high-dose cisplatin chemotherapy combined with radiotherapy in 60 patients affected by head and neck cancer. Oral nutritional supplements were used to reach the correct number of daily calories and proteins.

**Results and discussion:**

All patients completed radiotherapy as planned and the 96,4% of them did not interrupt chemotherapy for toxicity, reaching a total dose of at least 200mg/m2. Despite a rapid deterioration of body composition during treatment, nutritional support helped patients to maintain (or in some cases improve) anthropometric parameters from the end of chemoradiotherapy to the following 3 months. Low prealbumin and albumin pre-treatment led to higher risk of toxicities with consequent reduction of cisplatin dose intensity, whereas weight at the end of the treatment seems to be an interesting predicting factor for disease free and overall survival (p=0.007; p=0.015).

## Introduction

Head and neck cancers are a heterogeneous group of malignancies affecting the oral cavity, nasopharynx, oropharynx, hypopharynx, larynx, paranasal sinuses and salivary glands, which are challenging to treat due to their genetic complexity and aggressiveness. Globally, there are an estimated 890,000 new cases each year (about 4.5% of all cancer diagnoses) and 450,000 deaths (approximately 4.6% of global cancer deaths). Specifically, there are roughly 380,000 cases of lip and oral cavity cancers, 185,000 cases of laryngeal cancer, 133,000 cases of nasopharyngeal cancer, 98,000 cases of oropharyngeal cancer, 84,000 cases of hypopharyngeal cancer, and 54,000 cases of salivary gland cancer (1).

These cancers are primarily linked to tobacco use, alcohol abuse and viral infections, mainly Epstein–Barr virus and human papillomavirus (2). Other risk factors include radiation exposure, dietary patterns, alcohol-containing mouthwashes, poor oral hygiene and periodontal disease (3). The global distribution of risk factors affects the variability and incidence of these cancers, with worse prognosis for patients who receive a late diagnosis or have limited access to specialized care (4). For patients with locoregionally advanced head and neck squamous cell carcinoma (HNSCC) and unresectable tumors, or those with expected poor functional outcomes from surgery, primary concomitant chemoradiotherapy (CRT) with high-dose cisplatin (100 mg/m^2^) administered every three weeks for three cycles is the preferred treatment regimen (5–9). Moreover, the management is particularly complicated as some of these patients continue to use tobacco and alcohol during treatment, adversely affecting clinical outcomes (10).

Patients with locally advanced HNSCC often experience malnutrition, dysphagia and pain, leading to significant weight loss even before diagnosis (11, 12) and a loss of weight greater than 5% is an independent prognostic factor for poorer progression free survival (13). Malnutrition is associated with worse treatment outcomes, increased morbidity and mortality, treatment delays and unplanned hospitalizations (14). In several patients, malnutrition is present at diagnosis due to metabolic aberrations and difficulties with oral food intake, such as impaired swallowing or bolus passage, resulting in inadequate daily caloric intake. Therefore, nutritional support before, during and after treatment is crucial, even for overweight and obese patients whose high body mass index (BMI) might not initially suggest a risk for malnutrition (15). Additionally, nutritional counselling, which represents the first line of nutritional support, should be introduced before treatments begin to optimize body composition and address potential frailty (16, 17).

During treatments, patients frequently experience dysphagia, loss of appetite, fatigue, pain, dyspnea, xerostomia, sticky saliva and oral mucositis. These symptoms significantly impact nutritional status and the ability to maintain normal social relations, thereby affecting quality of life (18). Functional disturbances, such as issues with speech, swallowing, hearing and breathing, further complicate social interactions and contribute to physical and psychological difficulties, including changes in body image and loss of function (19).

The primary goal of nutritional support - provided before, during and after treatments - is to avoid weight loss and help patients complete the therapies with minimal toxicities (20). Cancer patients are often hypermetabolic compared to controls, making adequate nutrition critical to achieve this, oral nutritional supplements (ONS) help to ensure appropriate daily caloric and protein intake, as recommended by ESPEN guidelines (21).

This study aims to assess the impact of early and systematic nutritional counselling on the quality of life of patients with head and neck cancers, as well as on adherence to oncological treatments, tolerance, and overall response rates, with the goal of maintaining and potentially improving body composition.

## Materials and methods

This is an observational, prospective, single-center study. From May 2021 to March 2023, 60 patients were consecutively enrolled at IRCCS San Raffaele Hospital in Milan, Italy. Specifically, all adults aged ≥ 18 years with head and neck cancer and a histological diagnosis of squamous cell carcinoma of the oral cavity, oropharynx, nasopharynx, larynx, hypopharynx, or an unknown squamous cell primary of head and neck origin were included. These cases were discussed in a Head and Neck team meeting (composed of surgeons, medical oncologists, radiation oncologists, nutritionists, head and neck radiologists, nuclear medicine physicians, pathologists, odontologists) and were deemed medically fit for a treatment plan involving either primary or adjuvant radiotherapy with concurrent chemotherapy (high-dose cisplatin). Conversely, women known to be pregnant or planning to become pregnant during the trial period, as well as patients requiring total parenteral nutrition, were excluded from the study. Comparisons shown in this study refer to 3 time points: T0 (at the beginning of the treatment), T1 (at the end of the treatment) and T2 (3 months after the treatment), at each time point, laboratory tests were collected (in particular: complete blood count, creatinine, albumin and prealbumin) as well as data regarding nutritional assessment and intervention.

The study received approval from the Institutional Ethical Committee (San Raffaele Hospital, Milan, approval date 08/06/2022) and all patients provided informed consent. Data were collected in compliance with the approved guidelines and according to good clinical practice.

### Treatment

All patients were treated with helical TomoTherapy^®^ (HT-Accuray, Maddison, WI, USA).

In the radical setting, a 18-Fluorodeoxyglucose computed tomography (CT) positron emission tomography (PET) scan was performed to identify biological target volume (BTV). For these patients, a hypofractionated schedule with a simultaneous integrated boost was used: 54 Gy in 30 fractions to bilateral neck nodes and 66 Gy to the tumor and high risk/PET positive nodes. These patients received primary concomitant Cisplatin at 100 mg/m^2^ on day 1, 22, and 43 during standard fractionated radiotherapy (or on day 1 and 22 during accelerated radiotherapy) (7–9).

In the postoperative setting, radiotherapy was prescribed according to histological examination findings: 54 Gy in 30 fractions to low-risk volumes and 61.5-64 Gy in 30 fractions to high-risk volumes. These patients received Cisplatin at 100 mg/m^2^ on day 1, 22, and 43 during radiotherapy or a flat dose of 50 mg weekly. In all cases, patients received a total dose of at least 200 mg/m^2^ of Cisplatin (22–25). Toxicities of the treatment were scored according to Common Terminology Criteria for Adverse Events (version 4.03).

### Nutritional assessment and intervention

#### Nutritional risk screening

To evaluate the risk of malnutrition, Nutritional Risk Screening tool (NRS-2002) (26) and Mini Nutritional Assessment-Short Form tool (MNA-SF) (27) were used. For each patient, body weight and height were collected as well as the weight lost 3-6months before T0, BMI was calculated by dividing body weight (kg) by the height squared (m^2^), as described by the World Health Organization (28).

#### Body composition and functional assessment

A BIA 101 BIVA bioelectric impedance device (Akern^®^) was used to obtain the following parameters: standardized phase angle (SPA), body cellular mass index (BCMI), total body water (TBW, L), extracellular water (ECW, L), skeletal muscle mass (SM, kg), skeletal muscle index (SMI, kg/m^2^), appendicular skeletal muscle mass (ASMM, kg/m^2^) and fat mass index (FMI, kg/m^2^). BIVA, as a simple and non-invasive technique, estimates body composition by measuring the opposition (impedance) to an electrical current passing through the body. The impedance identifies cellular health and reflects membrane integrity, cell mass and hydration status (29). SPA was considered instead of Phase Angle to obtain measures independent from age, sex, ethnicity, and BMI (30).

Handgrip strength (HGS, kg) was assessed with a Camry Digital Hand Dynamometer (31) to determine muscle function and strength, this evaluation was considered together with data emerging from BIVA to identify patients at risk of sarcopenia and tailor nutritional counselling on that finding.

#### Appetite and quality of life

Patients reported their appetite levels using a visual analogue scale (VAS) to compare their sensations at different points in their therapeutic journey (32).

Quality of life was assessed using the Italian versions of FACT-H&N and EORTC QLQ-C35 questionnaires (33). The instruments consist of thirty-nine and thirty-eight questions, respectively, related to patient functioning and the severity of cancer-related symptoms. Final scores were calculated according to the scoring manual.

#### Nutritional requirements and intervention

Total daily energy requirements were calculated by multiplying the estimated resting energy expenditure (using the Harris‐Benedict equations) by a correcting factor of 1.5. The amount of ONS supplementation was set for each patient to reach calorie and protein needs as established by ESPEN guidelines (aiming for up to 25–30 kcal/kg/day and 1.5 g/kg/day of protein) (21), evaluating the specific food intake every three weeks from the beginning of chemoradiotherapy.

At each nutritional visit were considered symptoms such as dysgeusia, nausea, constipation and dysphagia to change consistency and possibly taste of the ONS, if needed to improve the compliance of the patients (maintaining the same nutritional values from a product to the respective alternative).

Nutritional counselling was conducted at the beginning of treatment and then every three weeks, selecting energy dense (up to 300-400 kcal/bottle) and/or high-protein ONS basing on each patient’s preferences and needs. The average protein content in the supplements was 20-40 g/day, adjusted to meet each patient’s estimated requirements and actual daily food intake. Due to rapid changes induced by the treatment, the supplementation plan was often adjusted every three weeks, taking into account alterations in body composition evaluated by BIVA.

Nutritional counselling included sample meal plans to create balanced breakfasts, lunches, dinners, and snacks with high concentrations of carbohydrates, proteins and fats to prevent the loss of weight, muscle mass and function. Meal plans were prepared in accordance with Italian Guidelines, in particular, were considered: “Linee Guida per una Sana Alimentazione” (Consiglio per la ricerca in agricoltura e l’analisi dell’economia agraria-Crea, 2018) and” IV Revisione dei Livelli di Assunzione di Riferimento di Nutrienti ed energia per la popolazione italiana-LARN” (Società Italiana di Nutrizione Umana-SINU, 2017). The plans were tailored to individual eating patterns and food preferences, with recipes suggestions to facilitate adherence.

When ONS were insufficient to meet nutritional goals or when oral intake of food or liquids by mouth was impossible due to treatment-related toxicities such as mucositis, artificial nutrition was initiated to prevent weight loss and subsequent malnutrition.

### Statistical analysis

Due to the exploratory nature of the study, no *a priori* sample size calculation and statistical power analysis were performed.

Comparisons of each variable at different time points were conducted using the Friedman test for repeated measures. Differences in categorical variables were analyzed using the χ^2^-test or Fisher’s exact test. Correlations were assessed with Spearman’s rank correlation coefficient. A generalized linear mixed model (GLMM) was used to compare changes in weight, with the FACT variable as the target variable. Disease free survival (DFS) and overall survival (OS) were estimated using the Kaplan-Meier method and compared with the log-rank test. Cox regression analysis was performed to estimate hazard ratio (HR).

All analysis were conducted using SPSS V.24 (IBM SPSS Statistics for Windows, Version 24.0. Armonk, NY, USA). Correlations were plotted with JMP^®^, Version 17 Pro. SAS Institute Inc., Cary, NC, 1989–2024. In all tests, a P-value <0.05 was considered significant. The Benjamini–Hochberg (B-H) procedure was applied to control the false discovery rate at 10% (34).

## Results

Sixty patients were consecutive enrolled and their characteristics are shown in **Table 1**.

**Table 1:** Characteristics of the patients.

Variables	Categories	Statistics	Values
Age (years)		N	60
		Median (min-max)	59.3 (27.2-78.9)
Gender	Female	N (%)	17 (28.3)
	Male	N (%)	43 (71.7)
BMI (kg/m²)		N	60
		Median (min-max)	25.3 (16.1-38.3)
Smoking	Non-Smoker	N (%)	9 (15.0)
	Ex-Smoker	N (%)	37 (61.7)
	Smoker	N (%)	14 (23.3)
Tumor site	Oropharynx	N (%)	24 (40.0)
	Mouth	N (%)	10 (16.7)
	Nasopharynx	N (%)	9 (15.0)
	Hypopharynx	N (%)	4 (6.7)
	Larynx	N (%)	3 (5.0)
	Other	N (%)	10 (16.7)
Stage	I	N (%)	2 (3.3)
	III	N (%)	8 (13.3)
	IV	N (%)	50 (83.3)
Potus	No	N (%)	25 (41.7)
	Yes	N (%)	35 (58.3)
Radiotherapy	Adjuvant	N (%)	18 (30.0)
	Radical	N (%)	42 (70.0)

All patients completed radiotherapy as planned and 96.4% did not interrupt chemotherapy due to toxicity, achieving a total dose of at least 200 mg/m^2^ (with 55.0% completing 300 mg/m^2^). Regarding OS, 73.3% of the patients were still alive in March 2024 and 65.0% were surviving without disease (**Table 2**). All the patients received tailored nutritional counselling with ONS at T0, T1 and T2.

**Table 2:** Clinical outcome of chemoradiotherapy.

	Total dose of cisplatin (mg/m^2^)		OS	DFS	NGT			PEG		
	200-275	300			T0	T1	T2	T0	T1	T2
**% of patients**	45.0	55.0	73.3	65.0	3.3	35.0	3.3	3.3	3.3	3.3

DFS, Disease Free Survival, NGT, Nasogastric tube, OS, Overall Survival, PEG, Percutaneous endoscopic gastrostomy.

A small number of patients started treatment (T0) with prior artificial nutrition. By the end of the six weeks (T1), 35.0% of patients were being fed with nasogastric tube and 3.3% with a percutaneous endoscopic gastrostomy. None of the patients placed percutaneous endoscopic gastrostomy during treatment (the 3.3% indicated in **Table 2** already had it at the beginning of the treatment), whereas some of them started at T0 with ONS and then placed nasogastric tube later when oral intake of food or liquids by mouth was impossible due to treatment-related toxicities.

**Figure 1:**
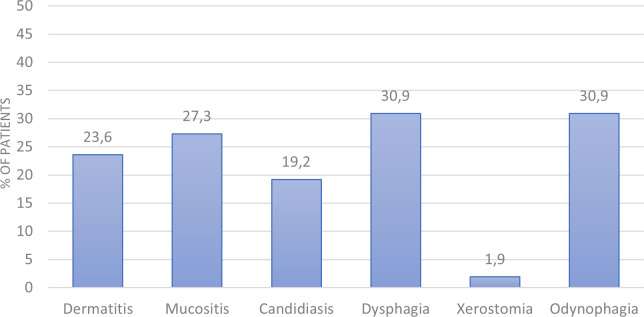
Toxicities during treatment.

The most frequent grade 3 toxicities experienced by patients during treatment are shown in **Figure 1**. The most common toxicities were odynophagia, dysphagia and mucositis (30.9%, 30.9% and 27.3%, respectively). Others included dermatitis, candidiasis and xerostomia (23.6%, 19.2% and 1.9%, respectively).

All collected variables at the start (T0) and end (T1) of CRT, as well as three months after (T2), are described in **Table 3**. The values are reported with median and interquartile range. Appetite, handgrip strength and nutritional risk did not deteriorate as rapidly as expected from T0 to T2. On the contrary, there was stability in VAS, HGS, MNA-SF and NRS-2002 scores from the beginning of the treatment to 3 months after.

**Table 3:** Variables from baseline to the end of treatment and 3 months after.

*Variables*	T0 (median, IQR) N=60	T1 (median, IQR) N=56	T2 (median, IQR) N=36
*BMI (kg/m^2^)*	25.4 (22.6-28.5)	23.5 (20.5-26.2)	23.3 (19.9-25.2)
*SPA*	0.17 (-0.6-0.8)	-0.3 (-1.2-0.3)	-0.5 (-1.1-0.1)
*BCMI*	10.2 (8.8-12.1)	9.2 (7.6-10.6)	9.3 (7.5-10.4)
*FFM (kg)*	57.9 (47.2-64.1)	53.1 (42.3-58.2)	53.7 (44.5-61.1)
*FFM (%)*	78.7 (74.0-83.5)	78.7 (72.7-84.3)	82.3 (77.6-85.7)
*FM (kg)*	15.4 (10.9-19.3)	14.6 (9.2-18.1)	11.7 (7.7-15.9)
*FM (%)*	21.4 (16.5-26.0)	21.3 (15.7-26.4)	17.7 (14.3-22.4)
*TBW (L)*	42.7 (34.7-47.2)	39.0 (31.1-42.8)	39.9 (32.9-45.0)
*ECW (L)*	19.9 (17.7-22.6)	18.8 (16.1-21.0)	19.9 (15.9-22.4)
*SM (kg)*	29.7 (22.6-31.9)	27.3 (19.5-29.3)	27.1 (22.4-30.7)
*SMI (kg/m^2^)*	10.0 (8.6-11.2)	9.1 (7.6-10.3)	9.7 (8.1-10.5)
*ASMM (kg)*	21.8 (17.8-24.3)	20.1 (15.5-22.3)	20.0 (16.3-23.1)
*FFMI (kg/m^2^)*	20.1 (17.8-22.3)	18.3 (16.2-19.9)	18.8 (16.3-21.2)
*FMI (kg/m^2^)*	5.7 (3.7-6.8)	5.3 (3.5-6.3)	4.1 (2.7-5.8)
*VAS (score)*	100.0 (62.5-100.0)	60.0 (30.0-100.0)	90.0 (60.0-100.0)
*HGS (kg)*	27.7 (20.7-40.3)	29.5 (21.6-36.3)	27.7 (22.1-34.8)
*MNA-SF (score)*	9.0 (8.0-11.0)	8.0 (6.0-10.0)	11.0 (9.0-12.0)
*NRS-2002 (score)*	3.0 (1.3-3.0)	3.0 (3.0-4.0)	3.0 (2.0-3.0)

ASMM, Appendicular Skeletal Muscle Mass, BCMI, Body Cell Mass Index, BMI, Body Mass Index, ECW, Extracellular Water, FFM, Fat-Free Mass, FFMI, Fat-Free Mass Index, FM, Fat Mass, FMI, Fat Mass Index, HGS, Handgrip Strength, MNA-SF, Mini Nutritional Assessment-Short Form, NRS, Nutritional Risk Screening, SM, Skeletal Muscle Mass, SMI, Skeletal Muscle Index, SPA, Standardized Phase Angle, TBW, Total Body Water, VAS, Visual Analogue Scale of Appetite.

Nutritional support helped patients maintain or, in some cases, improve their anthropometric parameters. For example, SMI increased from 9.1 kg/m^2^ at T1 to 9.7 kg/m^2^ at T2. While SM and SMI showed a significant decline from T0 to T1 (p=0.016 for both), this decline did not continue from T1 to T2 (p=1.000 for both). Data are presented in **Table 4**.

**Table 4:** Friedman test for repeated measures analysis of variance by ranks for anthropometric measure at T0, T1 and T2.

Variable	Sample1-Sample2	P-value
**BMI (kg/m^2^)**	BMI t2 - BMI t1	1.000
	BMI t2 - BMI t0	0.016*
	BMI t1 - BMI t0	0.016*
**SPA**	SPA t2 - SPA t1	1.000
	SPA t2 - SPA t0	0.016*
	SPA t1 - SPA t0	0.126
**BCMI**	BCMI t1 - BCMI t2	1.000
	BCMI t1 - BCMI t0	0.016*
	BCMI t2 - BMCI t0	0.016*
**FFM (kg)**	FFM t1 - FFM t2	1.000
	FFM t1 - FFM t0	0.016*
	FFM t2 - FFM t0	0.016*
**FFM (%)**	FFM% t1 - FFM% t0	1.000
	FFM% t1 - FFM% t2	0.846
	FFM% t2 - FFM% t0	1.000
**FM (kg)**	FM t2 - FM t1	1.000
	FM t2 - FM t0	0.016*
	FM t1 - FM t0	0.720
**FM (%)**	FM% t2 - FM% t0	1.000
	FM% t2 - FM% t1	0.846
	FM% t0 - FM% t1	1.000
**TBW (L)**	TBW t1 - TBW t2	1.000
	TBW t1 - TBW t0	0.016*
	TBW t2 - TBW t0	0.018*
**ECW (L)**		n.s
**SM (kg)**	SM t1 - SM t2	1.000
	SM t1 - SM t0	0.016*
	SM t2 - SM t0	0.432
**SMI (kg/m^2^)**	SMI t1 - SMI t2	1.000
	SMI t1 - SMI t0	0.016*
	SMI t2 - SMI t0	0.522
**ASMM (kg)**	ASMM t1 - ASMM t2	1.000
	ASMM t1 - ASMM t0	0.016*
	ASMM t2 - ASMM t0	0.016*
**FFMI (kg/m^2^)**	FFMI t1 - FFMI t2	1.000
	FFMI t1 - FFMI t0	0.016*
	FFMI t2 - FFMI t0	0.016*
**FMI (kg/m^2^)**	FMI t2 - FMI t1	1.000
	FMI t2 - FMI t0	0.016*
	FMI t1 - FMI t0	1.000
**VAS**	VAS t1 – VAS t2	1.000
	VAS t1 – VAS t0	0.522
	VAS t2 – VAS t0	1.000
**MNA-SF**	MNA t1 - MNA t0	0.612
	MNA t1 - MNA t2	0.016*
	MNA t2 - MNA t0	0.990
**NRS-2002**	NRS t2 - NRS t0	1.000
	NRS t2 - NRS t1	0.306
	NRS t0 – NRS t1	0.990

ASMM, Appendicular Skeletal Muscle Mass, BCMI, Body Cell Mass Index, BMI, Body Mass Index, ECW, Extracellular Water, FFM, Fat-Free Mass, FFMI, Fat-Free Mass Index, FM, Fat Mass, FMI, Fat Mass Index, HGS, Handgrip Strength, MNA-SF, Mini Nutritional Assessment-Short Form, NRS, Nutritional Risk Screening, SM, Skeletal Muscle Mass, SMI, Skeletal Muscle Index, SPA, Standardized Phase Angle, TBW, Total Body Water, VAS, Visual Analogue Scale of Appetite. * means p-value<0.05.

**Figure 2:**
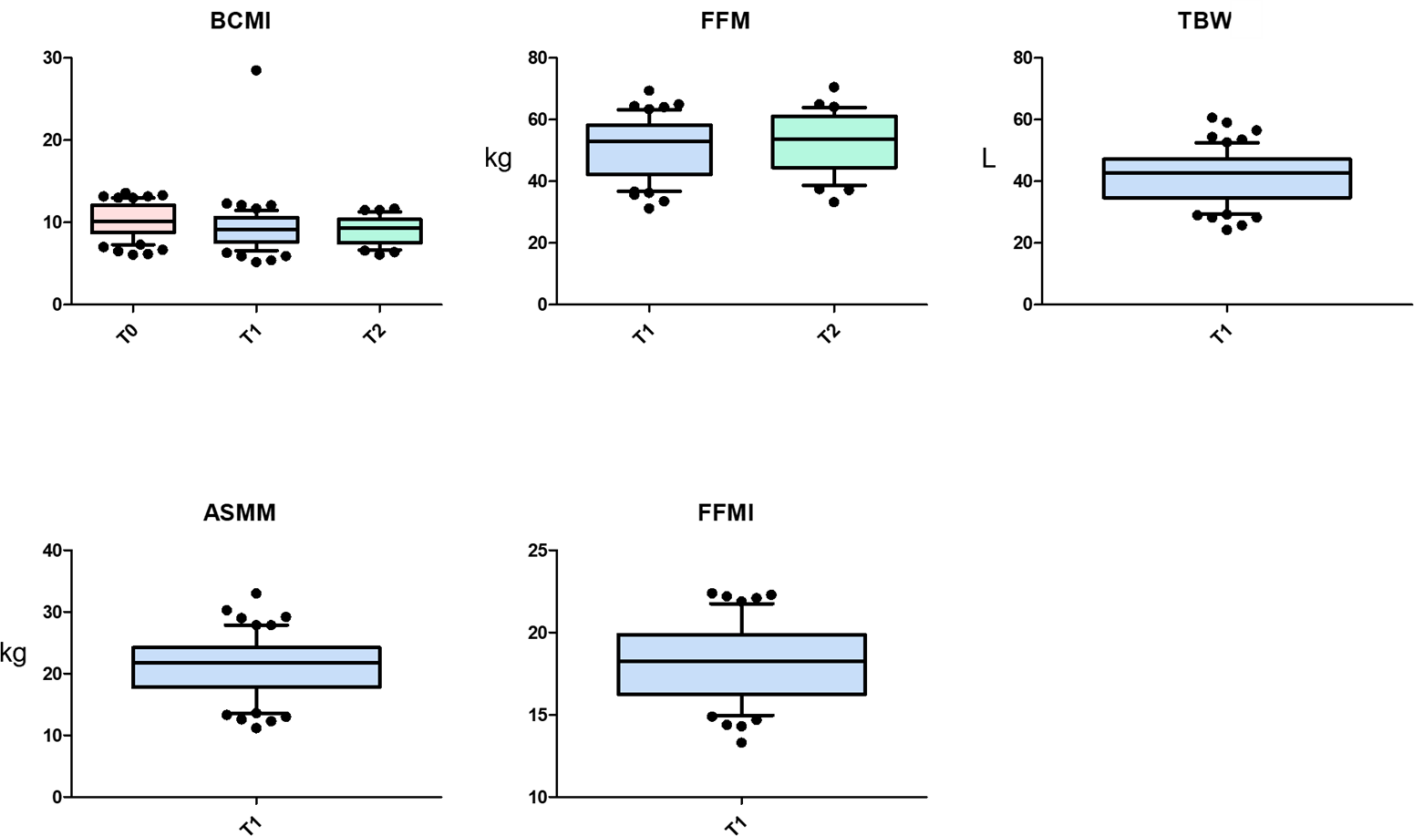
Correlations between pre-treatment prealbumin and anthropometric parameters. ASMM, Appendicular Skeletal Muscle Mass, BCMI, Body Cell Mass Index, FFM, Fat-Free Mass, FFMI, Fat-Free Mass Index, TBW, Total Body Water.

Low levels of prealbumin and albumin before treatment were associated with a higher risk of toxicities, which led to a reduction of cisplatin dose intensity. Pre-treatment prealbumin levels also showed a negative correlation with pre-treatment weight loss and several anthropometric parameters. As shown in **Figure 2**, pre-treatment prealbumin correlates with BCMI at T1 (p=0.016), FFM at T1 (p=0.020), TBW at T1 (p=0.037), ASMM at T1 (p=0.021) and FFMI at T1 (p=0.066). At T2, pre-treatment prealbumin correlates with BCMI (p=0.058) and FFM (p=0.097).

Quality of life questionnaires, FACTH&N and EORCT, showed a rapid deterioration in several variables from the beginning to the end of treatment, largely due to the severe side effects of CRT. However, there was an improvement in quality of life 3 months after treatment, in particular in areas such as sticky saliva, use of nutritional supplements, feeding tube use, weight loss/gain, pain, social eating, speech, coughing, open mouthing, dry mouth and swallowing.

The onset of toxicities was directly related to the dose intensity of cisplatin, as expected. However, there was no significant correlation between these toxicities and anthropometric measures or quality of life questionnaires at the end of CRT.

**Table 5** present the relationship between various variables (creatinine pre-treatment, sex, age, weight after treatment, weight loss before treatment, neutrophil-to-lymphocyte ratio at the end of treatment), DFS and OS. Although the number of patients limits the certainty of these findings, weight at T1 seems to be a significant predictor for DFS (p=0.007) and OS (p= 0.015).

**Table 5:** Cox multivariate analysis for DFS and OS.

		DFS			OS		
Variable	N	HR	95% C.I.	P value	HR	95% C.I.	P value
Creatinine pre-treatment							
**Below median**	27	1.089	0.398-2.983	0.868	2.818	0.701-	0.145
**Above median**	25	1			1	11.339	
Sex							
**Male**	37	0.844	0.285-2.499	0.760	1.040	0.271-3.987	0.954
**Female**	15	1			1		
Age							
**Below median**	27	2.183	0.773-6.162	0.143	1.702	0.483-6.002	0.408
**Above median**	25	1			1		
Weight at T1							
**Below median**	25	4.943	1.554-15.730	**0.007**	7.239	1.463-	**0.015**
**Above median**	27	1			1	36.819	
Weight loss median							
**Below median**	33	0.408	0.143-1.160	0.093	0.284	0.076-1.068	0.063
**Above median**	19	1			1		
NLR at T1							
**Below median**	27	0.703	0.225-1.938	0.496	0.597	0.175-2.037	0.410
**Above median**	25	1			1		

DFS, disease free survival; HR, hazard ratio; N, number of patients; NLR, neutrophil-to-lymphocyte ratio; OS, overall survival. Bold values indicate p-values <0.05.

## Discussion

Nutritional status is particularly important in HNSCC patients. The tumor itself can cause chewing difficulties, odynophagia, and dysphagia, leading to malnutrition. In addition, tooth extractions, usually performed before radiotherapy to minimize post-radiation complications or infections, further limit normal eating. Treatment side effects, especially from concomitant CRT, such as acute mucositis and long-term issues like dry mouth or sticky saliva, can also impair swallowing.

These challenges deeply affect patients’ impact body composition and quality of life, necessitating interventions to improve eating habits beyond treatment to prevent weight loss and reduced food intake. To mitigate malnutrition during CRT, prophylactic placement of a PEG tube might be considered. A randomized study showed that this approach resulted in fewer malnourished patients, longer enteral feeding and better quality of life 6 months after treatment without increased long-term dysphagia risk compared to standard clinical practice (35, 36). However, not all patients need enteral feeding, as seen in our study, where none required upfront PEG placement. Selecting high risk patients for malnutrition based on weight loss before treatment, age and radiotherapy dose to the constrictor muscles, can guide prophylactic PEG tube placement (37). Alternatively, nasogastric tube feeding effectively maintains body weight. In our Institution, this method is preferred due to its ease of placement and removal, as the optimal method for enteral feeding in HNSCC patients remain undetermined (38).

Protein-energy malnutrition can lead to sarcopenia, characterized by the loss of skeletal muscle mass and function (39). Sarcopenic patients with head and neck cancer have more than double the risk of severe treatment-related toxicity (40) and often require breaks in radiation treatment compared to non-sarcopenic patients (41). Dual-energy X-ray absorptiometry and magnetic resonance imaging are the gold standards for detecting low muscle mass but are limited in routine clinical practice due to high costs and radiation exposure concerns (42, 43). Another validated technique is CT-based body composition analysis at the third cervical vertebra (C3), which allow to identify the reduction of cervical muscles width (44).

For this study, we used BIVA and a dynamometer to detect sarcopenia, considering the strong relationship between isometric hand grip strength and lower extremity muscle power, knee extension torque and calf cross-sectional muscle area (45). These instruments are easy to use, inexpensive, reproducible and suitable for ambulatory patients.

Early intervention on sarcopenia with proactive exercise and nutritional counselling before treatment has been shown to reduce chemotherapy-related toxicity and improve OS, DFS and disease-specific survival (46). CRT strongly impacts food consumption, particularly affecting appetite and swallowing. ONS contribute to reach the nutritional requirements recommended by international guidelines (21). Patients benefit from high-quality protein sources, such as whey proteins, which are rapidly digested and increase plasma amino acid levels quickly (47). In cancer, protein breakdown is upregulated, leading to muscle weakness and dysfunction, but appropriate amounts of essential amino acids can preserve anabolic pathways and recovery (48). Whey protein supplementation improves body composition, muscle strength and treatment tolerance in malnourished advanced cancer patients, given their richness in cysteine, which supports glutathione synthesis and protects cells under chemotherapy-induced oxidative stress (49).

This study focused on the impact of nutritional counselling on treatment tolerance and outcomes. Our data showed that systematic nutritional counselling and regular body composition monitoring positively impacted on the onset of toxicities and significantly improved treatment adherence (100% completed radiotherapy, 96.4% chemotherapy). Although quality of life worsened during CRT, this trend was reversed within three months by continuing personalized nutritional counselling. Symptoms impacting the ability and desire to eat are common long-term issues for head and neck cancer survivors (50). Baseline-to-one-year changes in social eating problems are associated with swallowing-related quality of life, poor nutritional status, tumor site, age, and depressive symptoms (51). Early and systematic nutritional counselling is crucial for identifying and addressing patients’ critical issues from the start of oncologic treatment. As reported in several studies (52–54) and in our data, patients are frequently malnourished at diagnosis and treatment onset. Early nutritional intervention is facilitated by bioimpedance measurements at each nutritional visit, allowing immediate action if parameters worsen (e.g., BCMI and SPA). When ONS are insufficient to meet the energy requirements, nasogastric tubes are used for tailored enteral nutrition.

Serum albumin and prealbumin declines should be recognized as inflammatory markers rather than direct indicators of malnutrition. Indeed, visceral proteins decrease during the acute-phase response associated with acute and chronic illness and inflammation such as cancers and oncological treatments, regardless of underlying nutrition status, but normalization may indicate the resolution of inflammation, the reduction of nutritional risk, a transition to anabolism, and potentially lower calorie and protein requirements (55). In our study, prealbumin before CRT predicted body composition worsening at T1 and T2 and correlated with cisplatin dose intensity and treatment-related toxicities. Pre-treatment prealbumin was lower in patients with significant weight loss before CRT, likely due to its shorter half-life (2–3 days) compared to albumin (21 days).

The standard high-dose cisplatin regimen (100 mg/m^2^) for locally advanced squamous cell carcinoma of the head and neck often causes acute and late toxicities such as acute kidney injury (56), dysphagia, xerostomia, hypothyroidism, ototoxicity and osteoradionecrosis (57). In our study, dose reduction or treatment interruption was rare, and nutritional support was crucial to prevent and monitor complications, ensuring quality of life and body composition.

Finally, weight at the end of treatment emerged as a predictive factor for DFS and OS, suggesting that significant weight loss during treatment correlates with worsening health and slower recovery.

We deeply wanted to draw the attention on the changes in clinical outcome and quality of life of this set of patients when supported during the treatments by a multi-disciplinary approach that includes a nutritionist too, personalizing the pathway according to guidelines available for clinical nutrition (21). A similar customized structure is the key to ensure that the patients follow the instructions less laboriously and let them complete the set nutritional plan reducing the onset of toxicities related to chemoradiotherapy.

Our study’s limitations include a small sample size and the inability to evaluate average daily energy intake due to time constraints and the oncologic population’s linguistic/social issues. Future analysis aims to strengthen correlations between nutritional support and survival outcomes. Nutritional intervention was demonstrated, indeed, has been shown to improve three-year overall survival rates in head and neck cancer patients receiving nutritional counseling with/without ONS (58). Moreover, we considered a heterogeneous group of patients with different tumor types and stages, as well as confounding factors like artificial nutrition and surgery prior to treatment, which may influence chemoradiotherapy’s impact on body composition.

## Conclusion

Nutritional counselling before, during and after chemoradiotherapy significantly impact the quality of life of head and neck cancer patients and helps them to complete their prescribed treatments with a lower rate of toxicities. Close monitoring of body composition using BIVA enable the early intervention for weight loss with ONS or enteral nutrition. This approach prevents muscle reduction in function and strength by counteracting the catabolism associated with high-dose cisplatin and radiotherapy, ultimately allowing patients to complete their treatments while maintaining a better quality of life.

## Data Availability

The raw data supporting the conclusions of this article will be made available by the authors, without undue reservation.
